# Cytogenetic alterations in CML: not all created equal

**DOI:** 10.18632/oncotarget.24471

**Published:** 2018-02-10

**Authors:** Zimu Gong, Wei Wang, Shimin Hu

**Affiliations:** Shimin Hu: Department of Hematopathology, The University of Texas MD Anderson Cancer Center, Houston, Texas, USA

**Keywords:** chronic myeloid leukemia, BCR-ABL1, additional chromosomal abnormality, tyrosine kinase inhibitor, blast phase

The prognosis of patients with chronic myeloid leukemia (CML) has dramatically improved since the general availability of tyrosine kinase inhibitors (TKIs). Although uncommon, progression to blast phase (BP) remains a major concern in the management of patients with CML given the lack of effective treatment for and dismal outcome associated with BP. This necessitates the early identification of patients who are at high risk of developing BP so that a timely alternative treatment, such as allogeneic stem cell transplantation, may be offered before the onset of BP.

Additional chromosomal abnormalities (ACAs) are important determinants of outcome in CML patients, and are traditionally divided into major-route (+8, i(17q), +19, and +Ph) and minor-route (all other ACAs) subgroups based on their frequencies. In the 2013 European LeukemiaNet recommendations for the management of CML, major-route but not minor-route ACAs emerging during therapy are considered defining criteria for accelerated phase (AP) [1]. In the 2017 update of WHO Classification of Tumors of Hematopoietic and Lymphoid Tissue, all ACAs emerging during therapy are considered defining criteria for AP. In addition, major-route ACAs, complex karyotype and 3q26.2 abnormalities at initial diagnosis also define AP. With regard to the management, the emergence of major-route but not minor-route ACAs during therapy mandates the change of treatment. It is debatable whether this largely frequency-based stratification reflects the actual impact of each individual ACA and whether ACAs at initial presentation confer a prognostic value. Based on a large cohort of patients treated in the TKI era, we previously found that patients with isolated 3q26.2 rearrangements or -7/7q-, two minor-route ACAs, and patients with isolated i(17q) have a much poorer response to TKI treatment and a worse survival than patients with isolated +8 or +Ph, two major-route ACAs [2]. Additionally, the prognostic impact of +8, but not 3q26.2 rearrangement, is affected by the complexity of ACAs [2, 3]. Interestingly, once the disease progresses to the stage of BP, the type or the complexity of ACAs confers no prognostic value, supporting that the major role of ACAs lie in promoting BP [4, 5].

**Figure 1:**
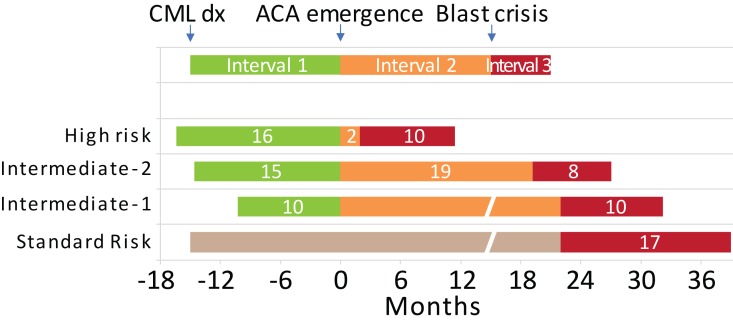
Schematic representation of disease course in CML patients with different risk types of additional chromosomal abnormalities

In a recent study, we investigated the impact of different type of ACAs on the disease progression of CML and found a significant difference in the latency from the emergence of ACAs to blastic transformation among different ACAs (Interval 2) [6]. Based on the difference, we establish a four-tier risk stratification model: the high-risk group includes patients with 3q26.2 rearrangement, -7/7q- or i(17q), either as an isolated single ACA or as a component of a complex karyotype. The intermediate-2 group includes patients with a complex karyotype but without any of the above-mentioned three high-risk components. The intermediate-1 group includes patients with any single ACAs other than the above-mentioned three high-risk ACAs. The standard risk group includes patients without ACAs (Figure 1). Interestingly, the median duration from initial diagnosis of CML to the emergence of ACA (Interval 1) is similar among three ACA risk groups, so is the medial survival after the onset of BP (Interval 3). The similarity of Interval-1 and Interval-3 among three ACA risk groups leaves the central determinant of CML disease course being the duration of Interval-2, which is highly ACA-dependent. In the pre-TKI era, most CML patients treated with busulfan or hydroxyurea developed BP within 2-4 years regardless of the karyotype, and the median survival after onset of BP was only 3-4 months. Our findings support that TKI therapy improves patient outcome through prolonging the duration of Interval 2. This four-tier stratification remains valid for ACAs detected at initial diagnosis of CML. Thus, based on the type of ACAs we are able to identify a subgroup of patients who are at a high risk of rapid blastic transformation and may benefit from early hematopoietic stem cell transplantation to prevent the onset of BP.

Answers to two other important cytogenetic questions start to emerge recently regarding to the significance of e1a2 (P190) *vs* e13a2/e14a2 (P210) subtype of *BCR-ABL1* and variant *vs* standard Philadelphia chromosome [7, 8]. We found that compared with patients with the typical e13a2/e14a2 *BCR-ABL1* transcript, patients with the e1a2 are more likely to present in BP initially, and those who do not present in BP initially have a higher risk of subsequent progression to BP, an inferior cytogenetic and molecular response to TKI therapy, and an inferior survival. Similarly, variant Philadelphia chromosome with four or more breakpoints, but not the balanced three-way translocations, confers a significant adverse prognostic value.

The current WHO criteria incorporate various parameters, including clinical and laboratory findings (counts of white blood cells, basophils, platelets and blasts, and splenomegaly), cytogenetic data (such as major-route ACAs at initial diagnosis and any ACAs acquired during therapy), and response criteria (such as ABL1 mutation), to define AP of CML. However, it has been shown that the impact of some of the clinical parameters has been minimized in the TKI era. Although implicated in the disease progression and adverse outcome in CML patients, not all ACAs or ABL1 mutations detected at diagnosis or acquired during therapy are equally prognostically significant in the TKI era. These findings indicate the need to revisit the current AP concept. From cytogenetic viewpoint, our four-tier risk stratification model provides a valuable alternative to the current binary stratification of chronic *vs* accelerated phase. Additionally, it may be worth taking into consideration of the e1a2 *BCR-ABL1* transcript subtype and variant Philadelphia chromosome among the ever-changing list of criteria for disease progression in a future classification scheme of CML.
